# Monitored Anesthesia Care Under a Combination of Low-Dose Remimazolam Infusion and Flumazenil Antagonism: A Case Report

**DOI:** 10.7759/cureus.46728

**Published:** 2023-10-09

**Authors:** Kotoe Kamata, Kenichi Masui

**Affiliations:** 1 Department of Anesthesiology and Perioperative Medicine, Tohoku University School of Medicine, Sendai, JPN; 2 Department of Anesthesiology, Yokohama City University School of Medicine, Yokohama, JPN

**Keywords:** pharmacokinetic simulation, monitored anesthesia care, conscious sedation and analgesia, flumazenil, remimazolam

## Abstract

Remimazolam is a novel benzodiazepine known for its short-acting properties. The safe use of flumazenil antagonism following remimazolam infusion remains a subject of debate. We present a case of monitored anesthesia care managed to be safe through low-dose remimazolam infusion and flumazenil antagonism. Pharmacokinetic simulations revealed that low-dose remimazolam was practically useful as one of the components of multimodal sedation/analgesia. Subsequent sedation with the readministration of remimazolam after a dose of flumazenil could also be attained.

## Introduction

Remimazolam is a novel benzodiazepine characterized by its short-acting properties with flumazenil as an antagonist [1]. However, the safe administration of the combination of remimazolam and flumazenil has been a subject of debate since a case of unanticipated resedation following flumazenil reversal after high-dose remimazolam infusion was reported [2]. In addition, there are no reports that the sedation-awake-sedation technique using flumazenil antagonism and readministration of remimazolam could provide the optimal level of consciousness for surgical intervention. We conducted a case of monitored anesthesia care (MAC) under low-dose remimazolam infusion and flumazenil antagonism. The time course of the effect-site concentration of each sedative/analgesic was calculated by a pharmacokinetic simulation to illustrate the drug interactions. This case report adheres to the Anaesthesia Case Report (ACRE) checklist derived from the ACRE guidelines [3].

## Case presentation

An 80-year-old woman (height: 152 cm; weight: 60 kg; American Society of Anesthesiologists physical status Class 3) was referred to our institution due to a complaint of progressively worsening hoarseness. Over the course of 10 years, an anterior mediastinal thymoma had exhibited progression unless treated with chemoradiotherapy. In response to superior vena cava syndrome, carotid artery stenting was performed as a palliative therapy. Hoarseness, attributed to left recurrent nerve palsy, had become apparent eight months before the surgery. Prior to the procedure, the patient's self-reported level of voice handicap was 56 by the Voice Handicap Index (VHI) [4]. The patient's comorbidities, including angina pectoris, hypertension, atrial fibrillation, and primary biliary cirrhosis were well managed with medications. The surgical plan involved thyroplasty type I and arytenoid adduction within the framework of laryngeal surgery for unilateral vocal fold paralysis, conducted under MAC. The Ethics Committee of Tohoku University School of Medicine approved this case presentation (No. 33875). The patient gave her informed consent for publication of this case report.

Supplemental oxygen was provided through a nasal cannula at 3 L/min, and peripheral oxygen saturation, end-tidal carbon dioxide, and respiratory rate were continuously monitored using a Capnostream® (Medtronic, Minneapolis, MN, USA). This was done along with monitoring the electrocardiogram, heart rate, and noninvasive blood pressure. Conscious sedation was initiated with remimazolam at 0.5 mg/kg/h and dexmedetomidine at 6 µg/kg/h. After seven minutes, the patient became unresponsive to verbal commands, and the infusion rates for remimazolam and dexmedetomidine were reduced to 0.25 mg/kg/h and 0.7 µg/kg/h, respectively. Infusion rates were adjusted to maintain a respiratory rate within the range of 10 to 20/min with normocapnia. Intravenous boluses of ketamine and fentanyl were administered to suppress responses to surgical stimuli.

Upon the otolaryngologist's request for patient awakening, remimazolam infusion was terminated 143 minutes after anesthesia induction. Dexmedetomidine administration was reduced from 0.5 µg/kg/h to 0.2 µg/kg/h. The patient rapidly regained consciousness within three minutes and could follow verbal commands without agitation. She did not even refuse nasoendoscopic observation. Although the patient was able to vocalize, dexmedetomidine infusion was terminated, and 0.2 mg of flumazenil was administered 155 minutes after induction. Two minutes later, a clear conversation was facilitated.

**Figure 1:**
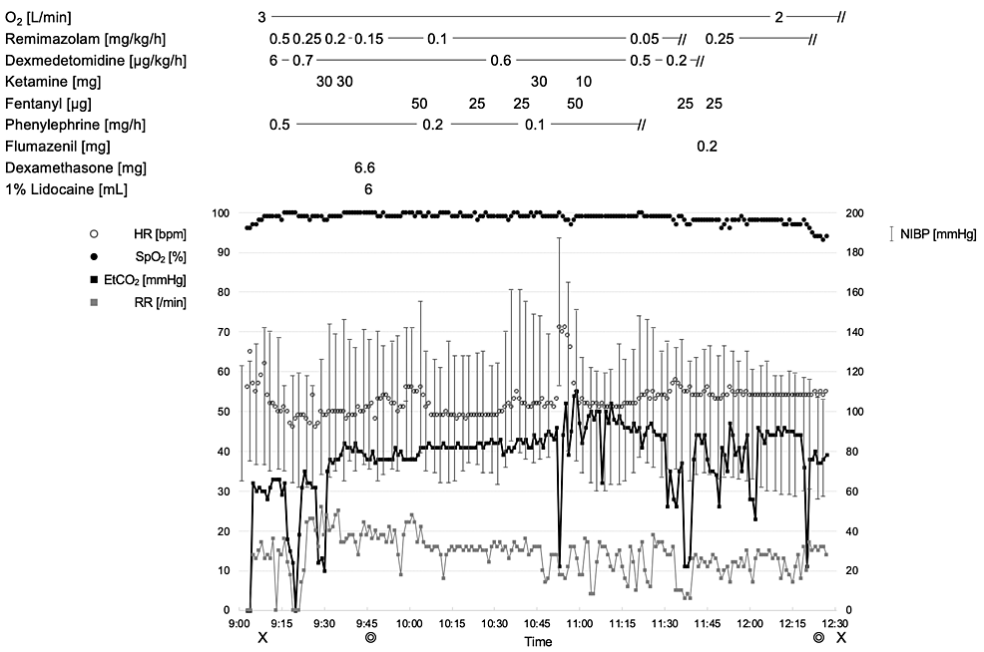
Intraoperative course. Anesthesia record of the current patient is shown. O_2_: oxygen, HR: heart rate, SpO_2_: arterial oxygen saturation, EtCO_2_: end-tidal carbon dioxide, RR: respiratory rate, NIBP: non-invasive blood pressure, X: anesthetic induction/end of anesthesia, ◎: start of operation/end of operation.

Surgical correction was completed under the patient's self-assessment of improved breathy hoarseness. Intravenous remimazolam was reintroduced at 0.25 mg/kg/h, beginning 161 minutes after induction, and maintained throughout wound closure. The patient regained consciousness thirteen minutes after remimazolam termination, and her cardiorespiratory status remained stable. A total of 28.2 mg of remimazolam and 128.8 µg of dexmedetomidine were administered. The total duration of the surgery and anesthesia was 160 minutes and 207 minutes, respectively. Figure 1 summarizes the anesthesia course. The patient's postoperative course was uneventful. Vocal training commenced on postoperative day (POD) 5 as planned. On POD 7, she was discharged from the hospital, expressing high satisfaction with the MAC provided. She reported neither unpleasant memories nor anxiety during the operation. At the six-month follow-up, she expressed satisfaction with a VHI score of 0.

For a detailed assessment of drug administration in this case, the remimazolam effect-site concentration (Ce), remimazolam equivalent Ce, flumazenil Ce, dexmedetomidine Ce, ketamine predicted plasma concentration (Cp), norketamine Cp, ketamine equivalent Cp, and fentanyl Ce were estimated using published pharmacokinetic model parameters [5-11]. The remimazolam equivalent Ce, calculated from the remimazolam and flumazenil Ce using published equations [12], accounts for the antagonistic effect of flumazenil on remimazolam. The ketamine equivalent Cp was determined as the sum of the ketamine Cp and one-third of the norketamine Cp [13]. This calculation considers norketamine as the main metabolite of ketamine, which, like ketamine itself, also possesses anesthetic properties.

## Discussion

This MAC case demonstrates the clinical utility of the combination of low-dose remimazolam and flumazenil. Under low-dose remimazolam-based MAC, the patient showed rapid recovery after flumazenil administration. Moreover, subsequent sedation with remimazolam successfully alleviated unpleasant memories and discomfort. An ideal MAC can flexibly adjust the patient's level of consciousness along with surgical requirements while maintaining a stable cardiorespiratory status. One of the most distinguishing features of remimazolam, compared to non-benzodiazepine intravenous sedatives such as propofol, is its susceptibility to antagonism by flumazenil.

Based on the pharmacokinetic simulation, Masui indicated that remimazolam overdose during anesthesia maintenance is a significant risk factor for the reappearance of an anesthesia/sedative effect of remimazolam after flumazenil reversal [12]. Even if the patient regains consciousness after a flumazenil bolus, the effects of remimazolam reappear if its residual concentration is high [2], because flumazenil antagonizes the effects of benzodiazepines via a competitive mechanism. The antagonistic effect of flumazenil is influenced by the concentrations of both flumazenil and benzodiazepines in proximity to benzodiazepine receptors. 

**Figure 2:**
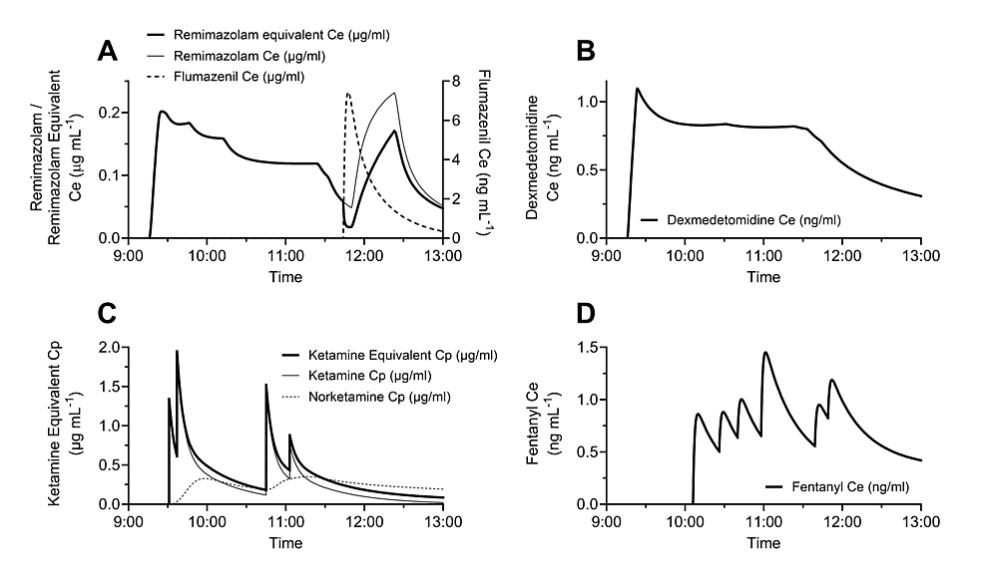
Time courses of anesthetic and opioid concentrations. A: Remimazolam effect-site concentration (Ce, thin line), remimazolam equivalent Ce (bold line), flumazenil Ce (dashed line), B: dexmedetomidine Ce, C: ketamine predicted plasma concentration (Cp, thin line), ketamine equivalent Cp (bold line), norketamine Cp (dashed line), and D: fentanyl Ce. The remimazolam equivalent Ce, which was calculated from remimazolam and flumazenil Ce using published equations [12], shows the remimazolam concentration taking into account the antagonistic effect of flumazenil. The ketamine equivalent Cp was calculated as the sum of the ketamine Cp and one-third of norketamine Cp, because norketamine is the main metabolite of ketamine, and, like ketamine itself, also has an anesthetic effect.

Regarding this issue, our choice of a low infusion rate for remimazolam is considered to have several clinical advantages, such as quick recovery and subsequent sedation. As shown in Figure 2, the effect-site concentration of remimazolam was kept at a low level at the end of initial sedation. Thus, a flumazenil bolus quickly elicited a clear conversation that enabled the patient to conduct a self-assessment for hoarseness. In addition, the surgical correction could be completed because she did not become drowsy. Furthermore, subsequent sedation could be managed by the readministration of remimazolam. The flumazenil dose of 0.2 mg was appropriate in this case because the readministration of remimazolam increased its equivalent Ce during the subsequent sedation (Figure 2A). Although pharmacokinetic simulations indicate that the Ce of all sedatives/analgesics, except remimazolam, decreased during this period, the patient was sufficiently sedated.

Low-dose remimazolam-based MAC has some issues that should be addressed. First, depending on the level of surgical intervention, multimodal sedation/analgesia is often necessary [14]. Our strategy involving the coadministration of dexmedetomidine is supported by a previous report. Gao et al. found that dexmedetomidine has a synergistic effect with remimazolam, resulting in reduced remimazolam doses during induction and maintenance [14]. Though their bronchoscopic observation was reported to be shorter and painless, intravenous sufentanil was given prior to examination [14]. In the present case, ketamine and fentanyl boluses were selected because prolonged immobilization of the patient and effective pain control were needed. Steep elevations of ketamine Cp and fentanyl Ce 108 minutes after induction were required to suppress coughing in response to arytenoid adduction (Figure 2). Second, the appropriate dose of flumazenil should be considered. The manufacturer's recommended dose of 0.2 mg was given to our patient under intensive cardiorespiratory observation. Pharmacokinetic simulations caution against the use of a higher dose of flumazenil due to the greater risk of the reappearance of remimazolam's sedative effect [12]. Third, the optimal rate of remimazolam infusion for subsequent sedation was not clarified. A lower infusion rate than in the general anesthesia regimen is considered favorable. To easily control the effect of remimazolam, a pharmacokinetic simulation is recommended using simulation software on a commercial anesthesia information management system or standalone software such as Tivatrainer (www.tivatrainer.com; Gutta B.V., Aerdenhout, The Netherlands), since a target-controlled infusion system is currently unavailable. Additionally, cardiorespiratory changes and the patient's responses must also be carefully observed.

## Conclusions

This case illustrates the feasibility of conducting MAC using a combination of remimazolam and flumazenil. Given the reappearance of the effects of remimazolam following reversal by flumazenil, low-dose remimazolam infusion with multimodal sedation/analgesia provides safe and effective MAC. Subsequent sedation after flumazenil antagonism can also be easily induced with the readministration of remimazolam.
